# P-1603. Is it Time to Abandon Intravascular Catheter Tip Cultures for the Diagnosis and Management of Central Line-Associated Bloodstream Infections (CLABSIs)?

**DOI:** 10.1093/ofid/ofae631.1770

**Published:** 2025-01-29

**Authors:** Ian Adrian F Frani, Justin Serfino, Alyssandra Marie Navarro

**Affiliations:** University Medical Center of Southern Nevada, Las Vegas, Nevada; University of the Philippines College of Medicine, Laveen, Arizona; University of Arkansas in Fayetteville, Hot Springs, Arkansas

## Abstract

**Background:**

The diagnostic and therapeutic utility of intravascular catheter tip cultures (CTCx) has been contested. The 2009 Infectious Diseases Society of America (IDSA) guidelines recommend a CTCx with blood cultures (BCx) to diagnose CLABSIs. Recent studies have demonstrated that BCx alone are sufficient to guide therapy. This study aims to evaluate the diagnostic and therapeutic utility of CTCx in CLABSIs.

**Figure 1.**
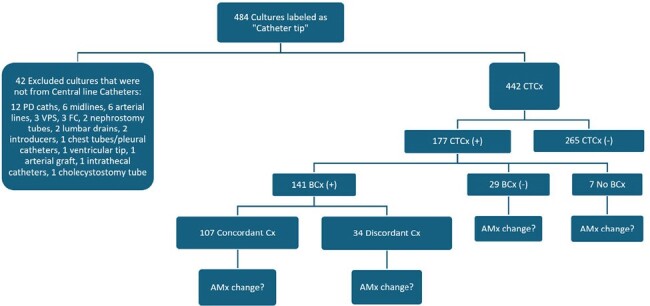
Summary of Catheter Tip Cultures and Corresponding Blood Cultures from 2017-2023 CTCx, Catheter tip cultures; (+) or (-), growth or no growth in cultures; BCx, blood cultures; CFU, colony forming units; AMx, antimicrobials; PD, peritoneal dialysis; VPS, ventriculoperitoneal shunts; FC, Foley catheters; Cx, cultures; Concordant Cx, at least 1 similar organism was isolated on both CTCx and BCx. Discordant Cx, CTCx, and BCx isolated different organism/s.

**Methods:**

We performed a retrospective chart review of patients with cultures labeled “Catheter tip” from 2017 to 2023. We looked at the concordance (similar organism/s isolated) or discordance (isolated different organism/s) of CTCx and BCx. We also conducted chart reviews to evaluate whether CTCx resulted in a change in antimicrobials (AMx).

**Table 1.**
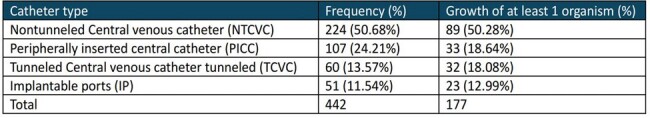
Classification of Intravascular Catheter Types and Culture Results

n=442, Total number of CTCx; n=177, Total number of CTCx with at least 1 organism isolated.

**Results:**

There were 442 CTCx out of 448 cultures collected (see Figure 1). Only 177 (40.05%) had ≥1 organism/s isolated. Out of 177 cases, 119 (67.23%) CTCx grew ≥15 colony forming units (CFU). About 58% (154/265) of negative CTCx had positive BCx. Table 1 listed the catheter types and the number of positive CTCx. The average time from BCx identification to catheter removal was 30 hours. Concordance was seen in 75.89% (107/141) and discordance in 24.11% (34/141). For all CTCx, concordance was present only in 24.21% (107/442). There were ≥15 CFU in 75.70% (81/107) of concordant cases, and 50% (17/34) in discordant cases. In CTCx with no BCx obtained or negative BCx, 58.33% (21/36) had ≥15 CFU. Figure 2 listed the organisms isolated on CTCx and Figure 3 showed the organisms isolated in concordant and discordant cases. CLABSIs were the source of bacteremia in 71.63% (101/141). In CTCx with positive BCx, CTCx results changed AMx in 6 cases (4.26%, 2 concordant, 4 discordant), no change in 132 cases (93.62%, 102 concordant, 30 discordant), and unclear in 3 cases. In CTCx with no BCx obtained or negative BCx, 30.56% (11/36) resulted in a change in AMx (10 with negative BCx and 1 without BCx) and was unclear in 1 case.

**Figure 2.**
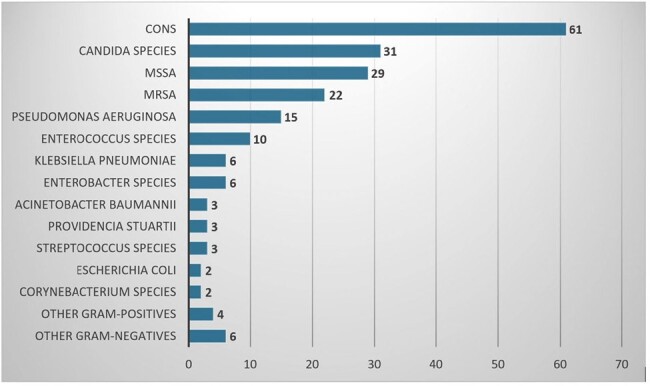
Organisms Isolated from Catheter Tip Cultures CONS, coagulase-negative staphylococcus; MSSA, methicillin-susceptible staphylococcus aureus; MRSA, methicillin-resistant staphylococcus aureus. Other gram-negatives: Elizabethkingia meningoseptica (1), Ochrobactrum anthropic (1), Serratia marcescens (1), Stenotrophomonas maltophilia (1), Proteus mirabilis (1), and Citrobacter freundii (1); Other gram-positives: Rothia mucilaginosa (1), Staphylococcus aureus, unspecified (3).

**Conclusion:**

Our study showed that CTCx didn’t result in a significant change in AMx. The majority of the CTCx were not able to identify any organisms. CTCx has limited utility for both diagnostic and therapeutic purposes when it comes to CLABSIs. We recommend the current IDSA criteria for diagnosing CLABSIs be reevaluated in favor of abandoning the practice of CTCx.

**Figure 3.**
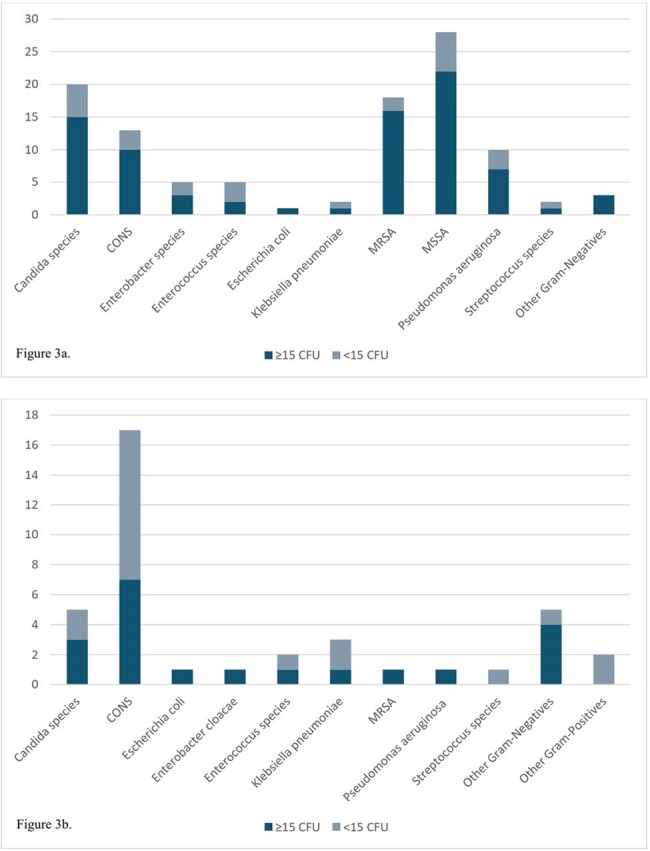
Organisms Isolated in Catheter Tip Cultures with Corresponding Blood cultures

Figure 3a. Organisms isolated in CTCx in concordant cases separated by CFU. Other Gram Negatives: Acinetobacter baumannii (1), Ochrobactrum anthropic (1), Serratia marcescens (1). Figure 3b. Organisms isolated in CTCx in discordant cases separated by CFU. Other Gram Negatives: Stenotrophomonas maltophilia (1), Providencia stuartii (2), Acinetobacter baumannii (1), Elizabethkingia meningoseptica (1); Other Gram Positives: Corynebacterium species (1), Staphylococcus aureus, unspecified (1).

**Disclosures:**

**All Authors**: No reported disclosures

